# Assessment and analysis of human laterality for manipulation and communication using the Rennes Laterality Questionnaire

**DOI:** 10.1098/rsos.170035

**Published:** 2017-08-23

**Authors:** Jacques Prieur, Stéphanie Barbu, Catherine Blois-Heulin

**Affiliations:** Ethos ‘Ethologie Animale et Humaine’, Université de Rennes 1 - CNRS UMR 6552, Station biologique de Paimpont, France

**Keywords:** gestural communication, manipulation action, body laterality, questionnaire, language lateralization, *Homo sapiens*

## Abstract

Despite significant scientific advances, the nature of the left-hemispheric systems involved in language (speech and gesture) and manual actions is still unclear. To date, investigations of human laterality focused mainly on non-communication functions. Although gestural laterality data have been published for infants and children, relatively little is known about laterality of human gestural communication. This study investigated human laterality in depth considering non-communication manipulation actions and various gesture types involving hands, feet, face and ears. We constructed an online laterality questionnaire including 60 items related to daily activities. We collected 317 594 item responses by 5904 randomly selected participants. The highest percentages of strong left-lateralized (6.76%) and strong right-lateralized participants (75.19%) were for manipulation actions. The highest percentages of mixed left-lateralized (12.30%) and ambidextrous (50.23%) participants were found for head-related gestures. The highest percentage of mixed right-lateralized participants (55.33%) was found for auditory gestures. Every behavioural category showed a significant population-level right-side bias. More precisely, participants were predominantly right-lateralized for non-communication manual actions, for visual iconic, visual symbolic, visual deictic (with and without speech), tactile and auditory manual gestures as well as for podial and head-related gestures. Our findings support previous studies reporting that humans have left-brain predominance for gestures and complex motor activities such as tool-use. Our study shows that the Rennes Laterality Questionnaire is a useful research instrument to assess and analyse human laterality for both manipulation and communication functions.

## 1. Introduction

Brain lateralization has been the subject of a substantial body of the literature for many years (e.g. see [1] for review). An increasing number of studies of different species support the hypothesis that behavioural lateralization would have been selected, because it provided significant advantages at both the individual and population levels [2,3]. First, lateralization would have appeared at the individual level because it enhanced brain efficiency [4]. Second, population-level lateralization would have emerged because it favoured social coordination between asymmetrical organisms (e.g. shoaling fish: [5]). Laterality patterns at the population level would be more prominent for social species than for solitary species (e.g. fish: [6]; tadpoles: [7]). Social pressures would thus have shaped laterality through natural selection, as recently supported by gestural studies (chimpanzees and gorillas: [8,9]; humans: [10]).

Human brains were reported to be laterally structured first for language-related functions [11]. Since then, neuroanatomical studies increasingly evidence that spoken language is lateralized in the left hemisphere (e.g. [12] for review). In addition, a close relationship between speech and gestures^1^ has been shown. Indeed, gestural communication involves brain regions similar to those processing spoken language (i.e. Broca's and Wernicke's areas, respectively, responsible for speech production and for understanding speech; [14,15]). Correlatively, reports evidence that manual gesture production involves the preferential use of the right hand. Studies concern deictic gestures such as POINTING^2^ and symbolic gestures produced by infants and children [16–18], undistinguished types of gestures accompanying speech by adult speakers [19–21] as well as sign language by deaf adult speakers [22–24]. Although left-brain specialization seems well admitted for gestures, only a few types of gestures have been considered. Furthermore, relatively few data are available for human adults. Therefore, to enhance our understanding of human laterality various types of gestures with large samples and many data points per subject must be taken into consideration.

The easiest observable laterality pattern of everyday life expressed by humans at the population level is the use of their right hand for distinct non-communication activities related to manipulation^3^ [25,26]. For example, 90% of individuals preferentially use their right hand for complex tasks such as writing, bimanual coordinated actions and tool use [27–29]. This right-handedness is consistent across time and across cultures, though the proportion of left-handed people varies from 0 to 27% [30–34]. This variation may be at least partly a consequence of disparities between methods used to assess manual laterality and of Laterality Index (LI) cut-offs used for categorizing individuals as ambidextrous, mixed right- or left-handed or strong right- or left-handed. A valid, reliable and fast way to assess the manual laterality of a large number of individuals is to implement a laterality questionnaire [35–38] and to apply the LI cut-offs criteria recently defined by Fagard *et al*. [39]. However, it must be noted that hand preference at both the individual and group levels measured by self-reported questionnaires has been shown to be potentially sensitive to the format of the questionnaire: the kind of items to be used [40], the type of answer choices (e.g. ‘either/no preference’: [41]), and the number of answer choices [35]. Studies also showed that hand preference measurement using questionnaires could be impacted by multiple factors [42,43], namely genetic factors [44,45], demographic factors such as age [46] and gender [47], cultural environmental factors such as forced right-hand use for writing (e.g. France: [48]; Finland: [44]), and region/area of the country [49], as well as performance abilities (e.g. mathematical ability: [50]; hand performance: [51]) and physical impairments (e.g. visual impairment: [52]). Altogether, these questionnaire studies emphasized the importance of carefully taking into account the multiple potentially influential factors on laterality, essential to avoid biases yielding ambiguous results and also a mandatory requirement to assess effects particular to the function (communication and non-communication).

Despite all the considerable scientific advances showing that humans predominantly exhibit a functional cerebral asymmetry towards the left for speech, gestures and manipulation, the nature of the relationships between language (speech and gestures) and handedness for manipulation has not yet been clearly elucidated, as emphasized by recent neuroimaging studies in adults [53–57]. For example, fMRI study of Häberling *et al*. [57] used factor analysis to identify the different systems underlying lateralized activities during three gestural tasks (observation of three distinct action sequences: pantomimes, sign language and dog performing movements) and two language tasks (a word generation task and a synonym task). Overall, they reported that the left hemisphere played the predominant role for both language and gesture observations. Results from their factor analysis of intercorrelations among handedness (assessed with a handedness inventory [58]) and laterality indices for gestural and language tasks suggest three independent networks, respectively, related to language, handedness and observation of manual actions independent of handedness. Further studies are necessary to investigate deeper the nature of the left-hemispheric systems involved in language and manual actions, in particular by taking into account a variety of communication and manual activities.

This study aimed to contribute to this research by investigating humans’ manual laterality for manipulation and various types of gestures (of which several of these have rarely or never been studied). To this end, we constructed a questionnaire to assess laterality for both non-communication and communication functions. As far as we know, this is the first questionnaire to address gestural laterality. We formulated a wide range of questions related to daily life manipulation and communication activities. We considered diverse behaviours involving hands, feet, face and ears and focused more particularly our attention on manual laterality in gestural communication. Here, we investigated laterality biases at the population level not only for manipulation actions but also for various types of gestures: iconic, symbolic, deictic (with and without speech), tactile and auditory gestures. Referring to the literature on human laterality, we predicted that most of these daily activities would be right-lateralized at the population level.

## 2. Material and methods

### 2.1. Participants

Questionnaire data were collected from April 2013 to December 2015: 5904 randomly selected participants responded to the French- and English-speaking versions of our questionnaire. However, because of incomplete questionnaire responses that did not meet the statistical criteria fixed for assessing laterality (see details below in Statistical analyses), we retained 5372 participants (see detailed numbers of participants per behavioural category in table 1). These 5372 participants' ages ranged from 6 to 84 years old (mean ± s.d., age = 35.34 ± 14.60). Our sample included 3372 women (62.77%; mean ± s.d., age = 34.49 ± 14.11), 1500 men (27.92%; mean ± s.d., age = 37.25 ± 15.50) and 500 participants (9.31%) who provided no personal information about their gender or age. As for previous questionnaire studies [39] considerably more women than men responded to our questionnaire. This is in accordance with previous findings showing that women are more inclined to participate in surveys than men [59].

**Table 1.** Characteristics, descriptive statistics and analyses of each behavioural category. Behavioural categories are classified by increasing LI values. *N* total: number of participants who responded to each behavioural category; data points total: number of data points associated with the *N* total participants; *N* analysed: number of participants who completed at least six items of each behavioural category; data points analysed: number of data points associated with *N* analysed; strong left: percentage of strong left-lateralized participants; mixed left: percentage of mixed left-lateralized participants; ambidextrous: percentage of ambidextrous lateralized participants; mixed right: percentage of mixed right-lateralized participants; strong right: percentage of strong right-lateralized participants; mean LI: mean laterality index score of *N* analysed, the sign indicates the direction of the behavioural bias (negative: left bias; positive: right bias); s.d. LI: Standard Deviation of the LI values; Wilcoxon test: *W*-value and *p*-value of the Wilcoxon test; mean ABSLI: mean absolute value of laterality index score of *N* analysed. Significant results are in bold.

behavioural category	*N* total	data points total	*N* analysed	data points analysed	strong left	mixed left	ambidextrous	mixed right	strong right	mean LI	s.d. LI	Wilcoxon test		mean ABSLI
*head-related gestures*	5904	40 973	5031	40 246	0.64	12.30	50.23	34.86	1.97	0.151	0.410	*W* = 2 937 819	***p *< 0.0001**	0.357
*manual gestures*														
visual deictic without speech	5904	38 146	5372	37 599	3.42	6.05	18.54	47.62	24.37	0.498	0.526	*W* = 11 789 530	***p *< 0.0001**	0.658
auditory	5904	38 131	5283	36 979	1.97	6.45	17.70	55.33	18.55	0.505	0.485	*W* = 11 794 431	***p *< 0.0001**	0.640
visual symbolic	5904	32 733	5065	30 390	2.90	6.40	11.31	52.00	27.39	0.537	0.507	*W* = 10 731 435	***p *< 0.0001**	0.675
tactile	5904	38 141	5182	36 268	0.29	5.06	19.78	54.69	20.18	0.538	0.419	*W* = 11 946 948	***p *< 0.0001**	0.617
visual iconic	5904	32 742	5114	30 684	2.19	7.12	10.44	48.49	31.76	0.564	0.505	*W* = 11 119 377	***p *< 0.0001**	0.696
visual deictic with speech	5904	34 979	4723	33 060	4.47	5.84	12.49	39.93	37.27	0.570	0.562	*W* = 9 378 145	***p *< 0.0001**	0.745
*podial gestures*	5904	31 839	4503	27 018	2.51	6.13	6.55	48.75	36.06	0.618	0.495	*W* = 8 858 473	***p *< 0.0001**	0.742
*non-communication* *manipulation actions*	5904	29 910	4910	29 460	6.76	3.75	1.65	12.65	75.19	0.754	0.579	*W* = 10 857 046	***p *< 0.0001**	0.934

In total, 4479 participants were from Europe (83.39%), 217 from the American continent (of which 196 from North America (3.65%), 21 from Central and South America (0.39%)), 110 from Africa (2.05%), 39 from Asia (0.73%), 20 from Oceania (0.37%). Five hundred and six participants (9.42%) gave no personal information about their current country of residence.

### 2.2. Questionnaire

Humans’ manual laterality for non-communication functions is commonly assessed via the following main handedness questionnaires: the Annett [60], Edinburgh [61], Healey [42] and Waterloo [43] instruments. So far, however, no questionnaire has allowed assessing human handedness for communication function. To study humans' manual laterality in both communication and non-communication functions, we thus designed an online laterality questionnaire including 60 items related to daily activities: 54 gestural communication items and six manipulation items (see the electronic supplementary material, S1 for the English-speaking version of the Rennes Laterality Questionnaire and electronic supplementary material, S2 for its French-speaking version). Gestural items included various behaviours involving hands (40), feet (6), face (6) and ears (2). We divided the 40 items on manual laterality for gestural communication into six categories: six iconic gestures (e.g. to describe a hilly landscape), six symbolic gestures (e.g. to indicate ‘no’ to someone), seven deictic gestures with speech (e.g. when saying to someone: ‘it's here’ by pointing the index finger to a place on a map to indicate where to go), seven deictic gestures without speech (e.g. to indicate a direction by pointing with your index finger), seven tactile gestures (e.g. to shake someone's hand) and seven auditory gestures (e.g. to applaud someone). Gestural items involving face and ears were grouped. The six items for manipulation were selected, because they were reported to elicit consistent individual hand preferences in most people and that they essentially elicit a right- or left-hand preference in self-reported right- or left-handed people [28,61]. To sum up, we considered the following nine categories: the six categories of manual gestures (40 items in all), the category of podial gestures (six items), the category of head (face/ear) gestures (eight items) and the category of manual manipulation (six items).

Guidelines for filling in our questionnaire were given at the beginning to help participants answer the questions appropriately (see the electronic supplementary material, S1 and S2). Participants were asked which body side they would use spontaneously to interact with a social partner or to manipulate an object. Four possible answers were proposed for each of the 60 items/questions: ‘Left’, ‘Right’, ‘Left or Right indifferently’ and ‘No reply’ (if he/she could not answer the question). We chose to add the ‘No reply’ response so that participants did not feel forced to answer a question either because he/she did not understand the question completely or because he/she did not know exactly his/her laterality related to the given item/question. In these cases, other responses would have introduced a bias in the questionnaire dataset. Furthermore, as previously mentioned, authors have suggested that humans' handedness can be impacted by multiple factors [42,43] such as social pressure (e.g. forced right-handedness evidenced in several countries for writing (e.g. France: [48]; Germany: [62]; Finland: [44]). Subsequently, we added 19 questions related to personal information (e.g. gender identity, age and current country of residence) to investigate the influence of multiple potential influential factors on human laterality (see the electronic supplementary material, S1 and S2). The study of the factors influencing human laterality for both non-communication and communication functions deserves to be investigated in depth using a comprehensive approach. Such multifactorial investigation is the subject of a current study [63].

### 2.3. Statistical analyses

For each behavioural category, only data for participants who completed at least six items of that category were included in the study to ensure more reliable assessment of laterality at both the participant and the category levels. We evaluated the direction of asymmetry of a given behavioural category (e.g. iconic gestures) for each participant by calculating an individual LI applying the formula LI = (number of right responses − number of left responses)/total number of responses that include all ‘Left’, ‘Right’ and ‘Left or Right indifferently’ responses. We estimated the associated strength of individual laterality by the absolute value of the LI (ABSLI) [39]). Evaluating LI and ABSLI by taking into account the trichotomous nature of laterality (i.e. considering not only the ‘Left’ and ‘Right’ responses, but also the ‘Left or Right indifferently’ responses) allows a more detailed and rigorous analysis of the direction and strength of laterality at both the participant and population levels and at both the item/question and category levels. In turn, it enables better discrimination of laterality both within and between participants and both within and between behavioural categories. The relevance of this formula is supported by a recent study of infant handedness [64]. Following Fagard *et al*. [39], we used [−100 to −90; −89.9 to −30; −29.9 to +30; +30.1 to +89.9; +90 to +100] as ranges of LI scores to classify participants as strong left-lateralized, mixed left-lateralized, ambidextrous, mixed right-lateralized and strong right-lateralized, respectively. We evaluated laterality bias at the population level for each behavioural category by a one-sample Wilcoxon signed-rank test because data did not fit a normal LI distribution.

Statistical analyses were computed using R v. 3.0.3 [65]. The level of significance was set at 0.05.

## 3. Results

We collected 317 594 item responses by 5904 participants between May 2013 and December 2015 (see the electronic supplementary material, table S1). After applying the statistical criteria fixed for assessing laterality, 301 704 item responses were retained for analyses (see detailed number of participants per category in table 1).

We analysed each of the nine behavioural categories separately to assess population-level laterality. The highest percentages of strong left-lateralized (6.76%) and strong right-lateralized participants (75.19%) were found for non-communication manipulation actions (table 1). The highest percentages of mixed left-lateralized (12.30%) and ambidextrous (50.23%) participants were found for head-related gestures. The highest percentage of mixed right-lateralized participants (55.33%) was found for auditory gestures. Statistical tests revealed that every behavioural category showed a significant right-side bias at the population level (one-sample Wilcoxon signed-rank tests, *p *< 0.0001; table 1).

## 4. Discussion

The goal of this study was to contribute to investigations of the nature of the left-hemispheric systems involved in language and manual actions. To do so, we studied humans' laterality for both non-communication and communication functions associated with diverse daily activities. Here, we questioned whether there is a laterality bias at the population level not only for non-communication manipulation actions, but also for various types of gestures (of which several had rarely or never been studied previously). Our results evidence that each of the nine behavioural categories we considered present a right-side bias at the population level. More precisely, participants were predominantly right-lateralized for non-communication manual actions, for visual iconic, visual symbolic, visual deictic (with and without speech), tactile and auditory manual gestures as well as for podial and head-related gestures.

To our knowledge, this study is the first to investigate human laterality biases at the population level not only for manipulation but also for various communication activities. Furthermore, this is the first study to address human laterality biases at the population level for podial gestures and for the following distinct manual gestures: visual iconic, visual symbolic, tactile and auditory. Our findings agree with previous reports for modern (Western and Westernized) societies showing a right-hand bias at the population level for distinct non-communication activities related to complex everyday manipulation tasks such as tooth brushing, tool using^4^ (e.g. hammering) and throwing [27,39,61]. Similarly, our findings are consistent with reports on manual gestures in modern societies evidencing right-hand biases at the population level for deictic gestures such as POINTING and symbolic gestures produced by infants and children [16–18], undistinguished types of co-speech gestures by adult speakers [19–21] and sign language by deaf adult speakers [22–24].

We also evidenced a right-side bias at the population level for head-related gestures, in agreement with previous reports for modern societies. Indeed, a right-side bias has been showed for kissing not only between interacting social partners (kissing: [68]; cheek kissing: [10]) but also between participants and a symmetrical doll's face to exclude any influence of the partner's turning preference [69,70]. Interestingly, Chapelain *et al*. [10] reported that laterality for cheek kissing (a frequent gesture in France used for greeting) varies in relation to the city considered suggesting that this behaviour could be subject to social pressures. It has also to be noted that their findings based on direct observations were consistent with their findings based on questionnaires and online surveys.

While ethological manual laterality studies in preliterate and preindustrial societies showed a strong right-hand use for tool-use tasks [31,34,71], their results reported that more commonplace object manipulation tasks (e.g. eating and holding) and non-object manipulation tasks (e.g. undistinguished types of gestures) appear to be far less lateralized. Further investigations of gestural laterality in preliterate and preindustrial societies are needed for a better understanding of the evolutionary origins of humans' left-cerebral lateralization for language (speech and gestures): first, by taking into account various distinguished types of gestures, second by considering as many potential influential factors as possible using a comprehensive approach.

Reports demonstrate that handedness is multidimensional not only for humans [42,43], but also for non-human primates [9,72]. For example, comprehensive multifactorial investigation of Prieur *et al*. [9] showed that chimpanzees' gestural laterality was influenced by several factors and their mutual intertwinement: gesture characteristics (sensory modality, use of a communication tool, sharing degree and gesture duration), interactional context (visual fields of both signaller and recipient as well as emotional context) and by individual sociodemographic characteristics of signaller and recipient (age, sex, group, hierarchy, affiliation and kinship). These findings set the stage for deeper investigations of human laterality in both non-communication and communication functions. Among other things, our questionnaire enhances our understanding of human laterality (i) by assessing the influence of several potential influential factors such as behavioural characteristics (e.g. gesture sensory modality: tactile, visual and auditory), interactional context (e.g. emotional valence) and sociodemographic characteristics (e.g. gender, age and current country and city of residence) and (ii) by further exploring the nature of the left-hemispheric systems for manipulation actions, gestural communication and other lateralized behaviours involving feet, face and ears. Such an in-depth investigation is the subject of a current study [63].

While authors argue that the use of a questionnaire is a valid and reliable way to investigate humans’ manual laterality considering a large number of individuals [35–38], other authors stress that assessments of hand preference via self-reported questionnaires can be potentially influenced by their associated format: the kind of items to be used [40], the type of answer choices (e.g. ‘either/no preference’: [41]) and the number of answer choices [35]. Therefore, a validation of the present questionnaire is planned and will be the subject of a future study.

To conclude, our study shows that humans present a right-side bias at the population level for daily activities, not only related to manipulation actions, but also to various types of gestures (of which several had rarely or never been studied previously). Indeed, the overwhelming majority of our participants from a large range of geographical, social and demographic backgrounds predominantly used their right-body side for various behaviours involving hands, feet, face and ears. Our findings thus provide additional support to previous studies reporting that humans have left-brain predominance for gestural communication and complex motor activities such as tool-use (e.g. see [73] for review). Our study shows that the Rennes Questionnaire is a useful research instrument to assess and to analyse human manual laterality for both manipulation and communication functions. Therefore, we propose that this questionnaire be used for future human laterality studies investigating both within- and between-population variations.

## Supplementary Material

ESM1. English-speaking version of the Rennes Laterality Questionnaire

## Supplementary Material

ESM2. French-speaking version of the Rennes Laterality Questionnaire

## Supplementary Material

Table S1. Raw data for each behavioural category

## Footnotes

1 From here on, the term ‘gesture’ is restricted to communication functions and defined as ‘movements of the limbs or head and body directed towards a recipient that are goal-directed, mechanically ineffective and receive a voluntary response’ ([13]; p. 4).

2 From here on, gestures are written in capitals.

3 From here on, we refer to manipulation as manual actions deprived of a communication function.

4 From here on, we define tool use as the movement of a detached object that is used to change the location or condition of another object or organism [66,67].

## Data accessibility

The datasets supporting this article have been uploaded as part of the electronic supplementary material.
