# Supplementary material for: Assessment and analysis of human laterality for manipulation and communication using the Rennes Laterality Questionnaire
Source: R Soc Open Sci. 2017 Aug 23;4(8):170035. doi: 10.1098/rsos.170035 (PMC5579081; doi:10.1098/rsos.170035)
Supplement: ESM1. English-speaking version of the Rennes Laterality Questionnaire [file rsos170035supp1.pdf]

To all this may concern,

Our research team (laboratoire ETHOS, CNRS-Université de Rennes 1) is currently carrying out an in depth study of human gestural communication.

We would gratefully appreciate your collaboration with this national and international study. You can help us by answering the following on-line questionnaire.

The questionnaire is a multiple-choice-questions (MCQ) survey that includes questions about various actions involving your hands, feet, ears and face.

The whole survey should take you about 10 minutes.

Looking forward to receiving your answers, we thank you very much in advance for your helpful collaboration.

Jacques Prieur, Catherine Blois-Heulin and Stéphanie Barbu, Université de Rennes 1 (France)

If you wish to receive more information or to know the results of this survey, do not hesitate to contact us:

Jacques Prieur jac.prieur@yahoo.fr

Catherine Blois-Heulin catherine.blois-heulin@univ-rennes1.fr

Stéphanie Barbu stephanie.barbu@univ-rennes1.fr

Section A: HAND

Instructions :

1 You must communicate by gesture ONLY, without uttering a word

2 Imagine that you interact with someone who is standing in front of you, facing you (except when the instruction says « with their back turned »)

A1. Imagine (if it helps, you can mime the gesture) which hand you would use spontaneously:

Choose the appropriate answer for each question:

	Left	Right	Left or Right indifferently	No reply
- to describe a hilly landscape to someone (with an undulating movement of your hand) ?	☐	☐	☐	☐
- to indicate « no » to someone (by wagging your index finger from one side to another) ?	☐	☐	☐	☐
- to indicate a direction by pointing with your index finger to show someone who is in front of you their way ?	☐	☐	☐	☐
- to shake someone's hand ?	☐	☐	☐	☐
- to applaud someone ? (which hand is above the other when you clap your hands ?)	☐	☐	☐	☐
- to indicate that your heart is beating too fast to someone (by miming rapid beats with your hand on your chest) ?	☐	☐	☐	☐
- to gesture « stop » to someone (by presenting your palm towards this person) ?	☐	☐	☐	☐
- to indicate that you have a headache (by putting your hand on your forehead) ?	☐	☐	☐	☐
- to punch an aggressor ?	☐	☐	☐	☐
- to bang your fist on the table when you are angry with someone ?	☐	☐	☐	☐
- to mime Pinocchio's long nose (by lengthening your nose with your hand) ?	☐	☐	☐	☐
- to show your approval to someone by giving them the « thumbs up » (by raising your thumb, the other fingers are bent) ?	☐	☐	☐	☐
- to show someone a star straight above you by pointing with your index finger ?	☐	☐	☐	☐
- to stroke a young child's head in sign of affection ?	☐	☐	☐	☐
- to beat the measure with a finger to give a music rhythm to someone ?	☐	☐	☐	☐

A2. Imagine (if it helps, you can mime the gesture) which hand you would use <u>spontaneously</u>:

Choose the appropriate answer for each question:

	Left	Right	Left or Right indifferently	No reply
- to give someone an idea of the height of an object (via the distance of your hand above the ground) ?	☐	☐	☐	☐
- to indicate that you wish someone good luck by crossing your index and middle fingers ?	☐	☐	☐	☐
- to signify the person you are talking to by pointing your index finger at this person ?	☐	☐	☐	☐
- to put your hand on someone's shoulder to comfort them ?	☐	☐	☐	☐
- to snap your fingers to attract attention from someone who is not listening ?	☐	☐	☐	☐
- to suggest to someone the shape of a square by moving your index finger to trace that shape ?	☐	☐	☐	☐
- to say « hello » to someone by waving your hand ?	☐	☐	☐	☐
- to indicate an object between you and the person you are talking to by pointing your index finger towards that object ?	☐	☐	☐	☐
- to tug on someone's clothes to draw their attention ?	☐	☐	☐	☐
- to knock on a window with one finger to draw someone's attention when this person is the other side of the window ?	☐	☐	☐	☐
- to give someone an idea of the width of a small object (via the distance between your thumb and your index finger) ?	☐	☐	☐	☐
- to make the "V sign" to someone (by raising the index and middle fingers and clenching the other fingers) ?	☐	☐	☐	☐
- to indicate to someone that you have a sore throat by putting your hand on your throat ?	☐	☐	☐	☐
- to make someone (who is moving away from you with their back turned) stop by grabbing the collar of their coat ?	☐	☐	☐	☐
- to tap your fingers on a table to signify someone your impatience ?	☐	☐	☐	☐
- to point to a dish placed between you and your pet when it is standing in front of you and facing you ?	☐	☐	☐	☐
- to stroke your pet when it is standing in front of you and facing you ?	☐	☐	☐	☐
- to call your pet by slapping your hand on your thigh ?	☐	☐	☐	☐

Section B: FOOT

Instructions :

1 You must communicate by gesture ONLY, without uttering a word

2 Imagine that you interact with someone who is standing in front of you, facing you

B1. Imagine (if it helps, you can mime the gesture) which foot you would use spontaneously:

Choose the appropriate answer for each question:

	Left	Right	Left or Right indifferently	No reply
- to play footsie with someone who is sitting at the same table facing you ?	☐	☐	☐	☐
- to stamp your foot on the ground to signify to someone that you are angry with them ?	☐	☐	☐	☐
- to kick a ball to send it to someone ?	☐	☐	☐	☐
- to kick an aggressor ?	☐	☐	☐	☐
- to tap your foot on the ground to signify to someone that you are impatient ?	☐	☐	☐	☐
- to approach the person you are talking to when you are standing still ? (which foot do you raise first ?)	☐	☐	☐	☐

Section C: FACE

Instructions :

1 You must communicate by gesture ONLY, without uttering a word

2 Imagine that you interact with someone who is standing in front of you, facing you (except when the instruction says « beside you »)

C1. Imagine (if it helps, you can mime the gesture) which side you would prefer spontaneously:

Choose the appropriate answer for each question:

	Left	Right	Left or Rignt indifferently	No reply
- to kiss someone's cheek ? (do you present first your left cheek or your right cheek ?)	☐	☐	☐	☐
- to hug someone when you meet the person again ? (when you hug, do you prefer that the head of the person is on your left or on your right ?)	☐	☐	☐	☐
- to sit beside someone who is sitting between two unoccupied chairs facing you in a waiting room ? (do you prefer to sit on the chair to your left or to your right ?)	☐	☐	☐	☐
- to avoid someone running towards you along a large path in a park ? (do you prefer the person to pass you on your left or on your right ?)	☐	☐	☐	☐
- to present your profile to someone who wants to take a photo of you ? (do you present you left or your right profile ?)	☐	☐	☐	☐
- to walk with a friend beside you ? (do you prefer your friend to be on your left or on your right ?)	☐	☐	☐	☐
- to listen to someone whispering ? (do you prick up your left ear or your right ear ?)	☐	☐	☐	☐
- to listen to someone talking to you on the telephone ? (do you place your phone near your left ear or your right ear ?)	☐	☐	☐	☐

Section D: HAND GESTURES WHILE TALKING

Instructions :

1 You must communicate by gesture ACCOMPANIED BY SPEECH

2 Imagine that you interact with someone who is standing in front of you, facing you

D1. Imagine (if it helps, you can mime the gesture) which hand you would use spontaneously:

Choose the appropriate answer for each question:

	Left	Right	Left or Right indifferently	No Reply
- when saying to help someone: « if you want my advice… » by accompanying the word « my » with a movement of your hand towards yourself ?	☐	☐	☐	☐
- when saying to someone: « you are going to lose a coat button » by pointing at that button with your index finger ?	☐	☐	☐	☐
- when saying to someone: « it's here » by pointing your index finger at a place on a map to indicate where to go ?	☐	☐	☐	☐
- when saying to someone who lies: « look me in the eyes ! » by pointing your index and middle fingers at your eyes ?	☐	☐	☐	☐
- when saying to someone: « you aren't careful, you've another spot on the middle of your sweater» by pointing at that spot with your index finger ?	☐	☐	☐	☐
- when saying to someone who is noisy: « stop you're giving me a headache » by pointing to your forehead with your hand ?	☐	☐	☐	☐
- when saying with anger: « come here!» to your pet by pointing at your feet with your index finger ?	☐	☐	☐	☐

Section E: HANDLING OBJECTS

E1. **Imagine (if it helps, you can mime the gesture) which hand you would use <u>spontaneously</u>:**

Choose the appropriate answer for each question:

	Left	Right	Left or Right indifferently	No Reply
- to use a hammer (to plant a nail)	☐	☐	☐	☐
- to use a spoon (to stir a liquid)	☐	☐	☐	☐
- to comb your hair	☐	☐	☐	☐
- to clean your teeth	☐	☐	☐	☐
- to use an eraser	☐	☐	☐	☐
- to throw a ball to reach a target	☐	☐	☐	☐

Section F: PERSONAL INFORMATION

F1. **How old are you?**

Please indicate your age here:

F2. **Are you male or female?**

Please select only one of the following propositions:

Female ☐

Male ☐

F3. In which country were you born?

Please inscribe your answer here:

F4. In which country, region (state for USA) and county did you spend your childhood?

Please inscribe your answer here:

Country

Region (State for USA)

County (if no equivalent administrative division, inscribe "No")

F5. In which city did you spend your childhood?

Please inscribe your answer here:

F6. In which country, region (state for USA) and county are you living now?

Please inscribe your answer here:

Country

Region (State for USA)

County (if no equivalent administrative division, inscribe "No")

F7. In which city are you living now?

Please inscribe your answer here:

F8. What socio-professional group do you belong to?

- Farmer ☐
- Tradesman, craftsman ☐
- Executive, manager, self-employed, private practitioner, freelance, intellectual ☐
- Technician or middle manager (administrative and commercial) in business, teaching, health care, public service ☐
- Employee, clerk, office worker ☐
- Labourer, worker ☐
- Student ☐
- Retired ☐
- No professional activity ☐

F9. What is your study level?

- Primary ☐
- Secondary (1st to 4th year) ☐
- Secondary (5th to 7th year) ☐
- Higher education (1st to 3rd year) ☐
- Higher education (4th to 5th year) ☐
- Higher education (>5th year) ☐

F10. Which hand do you use to write?

- Left ☐
- Right ☐

F11. Were you prevented from using your left hand during your childhood?

- Yes ☐
- No ☐

F12. Are any of your close relations left-handed?

Please choose the appropriate answer for each element:

	Yes	No	No answer
Father	☐	☐	☐
Mother	☐	☐	☐
Brother	☐	☐	☐
Sister	☐	☐	☐
Grand parents	☐	☐	☐
Your Father's brother or sister	☐	☐	☐
Your Mother's brother or sister	☐	☐	☐

F13. Which is your main guiding eye (the one for example you would use to look through a keyhole)?

Left ☐

Right ☐

F14. Have you any eyesight problems affecting your left eye?

	No problem	Slight	Medium	Strong
Myopia	☐	☐	☐	☐
Astigmatism	☐	☐	☐	☐
Presbyopia	☐	☐	☐	☐
Other problem	☐	☐	☐	☐

F15. **Have you any eyesight problems affecting your right eye?**

	No problem	Slight	Medium	Strong
Myopia	☐	☐	☐	☐
Astigmatism	☐	☐	☐	☐
Presbyopia	☐	☐	☐	☐
Other problem	☐	☐	☐	☐

F16. **Have you any hearing problems concerning your left ear?**

- My left ear presents no hearing loss ☐
- I have a slight hearing deficiency (mean loss between 20 and 40 dB) ☐
- I have a medium hearing deficiency (mean loss between 40 and 70 dB) ☐
- I have a severe hearing deficiency (mean loss between 70 and 90 dB) ☐
- I have a deep hearing deficiency (loss above 90 dB) ☐
- Other problems ☐

F17. **Have you any hearing problems concerning your right ear?**

- My right ear presents no hearing loss ☐
- I have a slight hearing deficiency (mean loss between 20 and 40 dB) ☐
- I have a medium hearing deficiency (mean loss between 40 and 70 dB) ☐
- I have a severe hearing deficiency (mean loss between 70 and 90 dB) ☐
- I have a deep hearing deficiency (loss above 90 dB) ☐
- Other problems ☐

F18. **Have you any cervical problems?**

- I have no cervical problems ☐
- Turning my head to the left causes some pain/discomfort in my neck ☐
- Turning my head to the right causes some pain/discomfort in my neck ☐
- Turning my head both to the left and to the right causes some pain/discomfort in my neck ☐

F19. Have you any back problems?

- [ ] I have no back problems
- [ ] Turning my chest to the left causes some pain/discomfort
- [ ] Turning my chest to the right causes some pain/discomfort
- [ ] Turning my chest both to the left and to the right causes some pain/discomfort

F20. Which hand do you use to write?

- [ ] Left
- [ ] Right
