# Supplementary material for: Assessment and analysis of human laterality for manipulation and communication using the Rennes Laterality Questionnaire
Source: R Soc Open Sci. 2017 Aug 23;4(8):170035. doi: 10.1098/rsos.170035 (PMC5579081; doi:10.1098/rsos.170035)
Supplement: ESM2. French-speaking version of the Rennes Laterality Questionnaire [file rsos170035supp2.pdf]

Bonjour,

Nous effectuons une étude sur la gestualité chez l’humain au sein de l'Unité de Recherche EthoS (Université de Rennes1/CNRS).

Nous sollicitons votre aide pour cette étude nationale et internationale.

Vous êtes invités à remplir le questionnaire ci-après.

Le questionnaire est un QCM qui comprend des questions sur diverses actions impliquant les mains, les pieds, les oreilles et le visage.

Il faut une dizaine de minutes pour remplir le questionnaire.

Merci beaucoup d’avance pour votre précieuse contribution !

Jacques Prieur, Catherine Blois-Heulin et Stéphanie Barbu, Université de Rennes 1 (France)

Si vous souhaitez plus d’informations, ou connaitre les résultats de cette étude, n’hésitez pas à nous contacter:

Jacques Prieur jac.prieur@yahoo.fr

Catherine Blois-Heulin catherine.blois-heulin@univ-rennes1.fr

Stéphanie Barbu stephanie.barbu@univ-rennes1.fr

Partie A: La main

Consignes :

Vous communiquez <u>par geste UNIQUEMENT (sans parole)</u> Imaginez que vous interagissez avec quelqu'un qui se trouve <u>debout, face à vous, le visage tourné vers vous</u> (sauf indication contraire mentionnée par « en vous tournant le dos »).

A1. Imaginez (si besoin faites le geste), quelle main utilisez-vous **<u>spontanément</u>** pour:

	Gauche	Droite	Gauche ou droite indifférent	Pas de réponse
- décrire un paysage vallonné à quelqu'un (par un mouvement d'ondulation de votre main) ?	☐	☐	☐	☐
- faire le geste de négation « non » à quelqu'un (en balançant votre index d'un côté et de l'autre) ?	☐	☐	☐	☐
- montrer du doigt à quelqu'un une direction qui se trouve en face de vous pour lui indiquer son chemin (en pointant votre index) ?	☐	☐	☐	☐
- serrer la main de quelqu'un que vous rencontrez ?	☐	☐	☐	☐
- applaudir quelqu'un ? (quelle main se trouve au-dessus de l'autre quand vous applaudissez ?)	☐	☐	☐	☐
- signifier que votre cœur bat très fort à quelqu'un (en faisant un geste de battement rapide avec votre main au niveau du thorax) ?	☐	☐	☐	☐
- faire le geste « stop » à quelqu'un (en dirigeant la paume de votre main vers cette personne) ?	☐	☐	☐	☐
- indiquer à quelqu'un que vous avez mal à la tête (en posant votre main sur votre front) ?	☐	☐	☐	☐
- donner un coup de poing à un agresseur ?	☐	☐	☐	☐
- taper du poing sur la table lorsque vous êtes en colère après quelqu'un ?	☐	☐	☐	☐
- mimer le long nez de Pinocchio (en prolongeant votre nez avec votre main)	☐	☐	☐	☐
- faire le « pouce levé » en signe d'approbation à quelqu'un (en dirigeant votre pouce vers le haut, les autres doigts sont repliés) ?	☐	☐	☐	☐
- montrer du doigt à quelqu'un une étoile qui se trouve au-dessus de vous (en pointant votre index vers l'étoile) ?	☐	☐	☐	☐
- caresser les cheveux d'un jeune enfant en signe d'affection ?	☐	☐	☐	☐
- battre la mesure avec le doigt pour donner le rythme d'une musique à quelqu'un ?	☐	☐	☐	☐

A2. Imaginez (si besoin faites le geste), quelle main utilisez-vous **<u>spontanément</u>** pour:

	Gauche	Droite	Gauche ou droite indifférent	Pas de réponse
- donner à quelqu'un une idée de la hauteur d'un objet (via la distance entre votre main et le sol) ?	☐	☐	☐	☐

	Gauche	Droite	Gauche ou droite indifférent	Pas de réponse
- signifier que vous souhaitez bonne chance à quelqu'un en croisant les doigts de votre main (index et majeur) ?	☐	☐	☐	☐
- désigner votre interlocuteur (en pointant votre index sur lui) ?	☐	☐	☐	☐
- poser la main sur l'épaule de quelqu'un pour le réconforter ?	☐	☐	☐	☐
- claquer des doigts pour attirer l'attention de quelqu'un qui n'écoute pas?	☐	☐	☐	☐
- suggérer à quelqu'un la forme d'un carré (en déplaçant votre index pour décrire cette forme) ?	☐	☐	☐	☐
- dire bonjour à quelqu'un (en agitant votre main) ?	☐	☐	☐	☐
- indiquer à quelqu'un un objet se trouvant entre vous et votre interlocuteur (en pointant votre index sur cet objet) ?	☐	☐	☐	☐
- tirer le vêtement de quelqu'un pour attirer discrètement son attention ?	☐	☐	☐	☐
- frapper à une vitre avec un doigt de votre main pour attirer l'attention de quelqu'un qui se trouve de l'autre côté de la vitre ?	☐	☐	☐	☐
- donner à quelqu'un une idée de l'épaisseur d'un petit objet (via l'écartement entre votre pouce et votre l'index) ?	☐	☐	☐	☐
- indiquer le « V » de la victoire à quelqu'un (en écartant l'index et le majeur de votre main, les autres doigts sont repliés) ?	☐	☐	☐	☐
- indiquer à quelqu'un que vous avez mal à la gorge (en posant votre main sur votre gorge) ?	☐	☐	☐	☐
- forcer à s'arrêter quelqu'un qui s'éloigne de vous, en vous tournant le dos, en saisissant le col de son vêtement ?	☐	☐	☐	☐
- tapoter des doigts sur la table pour signifier votre impatience à quelqu'un ?	☐	☐	☐	☐
- pointer du doigt une gamelle qui se trouve entre vous et votre animal de compagnie (votre animal se trouve debout, en face de vous, la tête étant face à vous) ?	☐	☐	☐	☐
- caresser votre animal de compagnie qui se trouve debout, en face de vous, la tête étant face à vous ?	☐	☐	☐	☐
- faire venir votre animal de compagnie en tapant votre main sur votre cuisse ?	☐	☐	☐	☐

Partie B: Le pied

Consignes:

Vous communiquez par geste UNIQUEMENT (sans parole) Imaginez que vous interagissez avec quelqu'un qui se trouve debout, face à vous, le visage tourné vers vous.

B1. Imaginez (si besoin, faites le geste), quel pied utilisez-vous spontanément pour :

	Gauche	Droite	Gauche ou Droite indifférent	Pas de réponse
- faire du pied à quelqu'un (cette personne est assise à table en face de vous) ?	☐	☐	☐	☐
- taper du pied sur le sol pour manifester votre colère envers quelqu'un ?	☐	☐	☐	☐
- taper dans un ballon pour l'envoyer à quelqu'un ?	☐	☐	☐	☐
- donner un coup de pied à un agresseur ?	☐	☐	☐	☐
- tapoter du pied sur le sol pour signifier votre impatience à quelqu'un ?	☐	☐	☐	☐
- vous approcher d'un interlocuteur alors que vous êtes à l'arrêt ? (quel pied avancez-vous en premier ?)	☐	☐	☐	☐

Partie C: Le visage

Consignes :

Vous communiquez par geste UNIQUEMENT (sans parole) Imaginez que vous interagissez avec quelqu'un qui se trouve debout, face à vous, le visage tourné vers vous (sauf indication contraire mentionnée par « en vous tournant le dos »).

C1. Imaginez (si besoin faites le geste), de quel côté préférez-vous spontanément:

	Gauche	Droite	Gauche ou Droite indifférent	Pas de réponse
- tendre votre joue vers votre ami pour lui faire la bise ? (présentez-vous d'abord votre joue gauche ou votre joue droite ?)	☐	☐	☐	☐
- serrer quelqu'un dans vos bras lors de retrouvailles ? (lorsque votre tête est près de la sienne, préférez-vous que sa tête soit à votre gauche ou à votre droite ?)	☐	☐	☐	☐
- vous asseoir à côté de quelqu'un assis entre deux chaises libres dans une salle d'attente, les chaises tournées vers vous? (préférez-vous vous asseoir sur la chaise à votre gauche ou à votre droite ?)	☐	☐	☐	☐
- éviter quelqu'un qui arrive en courant en face de vous sur une très large allée d'un parc? (pour le contourner, préférez-vous qu'il soit à votre gauche ou à votre droite ?)	☐	☐	☐	☐

	Gauche	Droite	Gauche ou Droite indifférent	Pas de réponse
- présenter votre visage à quelqu'un qui vous photographie ? (lui présentez-vous le côté gauche de votre visage ou le côté droit ?)	☐	☐	☐	☐
- marcher à côté de votre ami (préférez-vous qu'il soit à votre gauche ou à votre droite ?)	☐	☐	☐	☐
- tendre l'oreille vers quelqu'un qui parle à voix basse ? (tendez-vous l'oreille gauche ou l'oreille droite?)	☐	☐	☐	☐
- écouter votre correspondant parler au téléphone ? (placez-vous l'écouteur sur votre oreille gauche ou votre oreille droite?)	☐	☐	☐	☐

Partie D: La main avec la parole

Consignes :

Vous communiquez maintenant par geste ACCOMPAGNE PAR LA PAROLE Imaginez que vous interagissez avec quelqu'un qui se trouve debout, face à vous, le visage tourné vers vous.

D1. Imaginez (si besoin faites le geste), quelle main utilisez-vous spontanément:

	Gauche	Droite	Gauche ou Droite indifférent	Pas de réponse
- en disant à quelqu'un pour l'aider "si vous voulez mon avis" lorsque vous accompagnez le mot "mon" d'un geste de cette main vers vous ?	☐	☐	☐	☐
- en disant à quelqu'un "tu vas perdre ton bouton de manteau" lorsque vous pointez ce bouton avec votre index?	☐	☐	☐	☐
- en disant à quelqu'un "c'est ici" lorsque vous pointez votre index sur un endroit de la carte pour lui indiquer où aller?	☐	☐	☐	☐
- en disant à quelqu'un qui vous ment "regarde-moi dans les yeux!" lorsque vous pointez votre index et votre majeur sur vos yeux ?	☐	☐	☐	☐
- en disant à quelqu'un "tu n'es pas soigneux, tu as encore fait une tache au milieu de ton pull" lorsque vous pointez cette tache avec votre index ?	☐	☐	☐	☐
- en disant à quelqu'un de bruyant "arrête, tu me fais mal à la tête" lorsque vous pointez votre front avec votre main ?	☐	☐	☐	☐
- en disant avec colère " viens ici!" à votre animal de compagnie lorsque vous pointez vos pieds avec votre index ?	☐	☐	☐	☐

Partie E: Manipulation d'objets

E1. Imaginez (si besoin faites le geste), quelle main utilisez-vous spontanément:

	Gauche	Droite	Gauche ou Droite indifférent	Pas de réponse
- pour utiliser un marteau (pour enfoncer un clou)	☐	☐	☐	☐
- pour utiliser une cuillère (pour remuer un liquide)	☐	☐	☐	☐

	Gauche	Droite	Gauche ou Droite indifférent	Pas de réponse
- pour vous coiffer avec un peigne	☐	☐	☐	☐
- pour vous brosser les dents	☐	☐	☐	☐
- pour effacer avec une gomme	☐	☐	☐	☐
- pour lancer une balle en vue d'atteindre une cible	☐	☐	☐	☐

Partie F: Informations personnelles

F1. **Quel âge avez-vous ?**

F2. **Etes-vous de sexe féminin ou masculin ?**

Féminin ☐

Masculin ☐

F3. **Quel est votre pays d'origine ?**

F4. **Quel est votre département d'origine ? (celui où vous avez vécu pendant l'enfance)**

F5. **Quelle est votre ville d'origine (celle où vous avez vécu pendant l'enfance)**

F6. **Quel est le département où vous habitez actuellement ?**

F7. **Quelle est la ville où vous habitez actuellement ?**

F8. Quelle est votre catégorie socio-professionnelle ?

- Agriculteurs exploitants ☐
- Artisans, commerçants ☐
- Cadres, professions libérales et intellectuelles ☐
- Techniciens et professions intermédiaires (administratives et commerciales) des entreprises, enseignement, santé, fonction publique ☐
- Employés ☐
- Ouvriers ☐
- Elèves, étudiants ☐
- Retraités ☐
- Sans activité professionnelle ☐

F9. Quel niveau d'étude avez-vous ?

- Primaire ☐
- Secondaire (1ère à 4ème année) ☐
- Secondaire (5ème à 7ème année) ☐
- Etudes supérieures (1 à 3 ans d'études supérieures) ☐
- Etudes supérieures (4 à 5 ans d'études supérieures) ☐
- Etudes supérieures (plus de 5 ans) ☐

F10. Quelle est votre main d'écriture ?

- Gauche ☐
- Droite ☐

F11. Vous a-t-on empêché d'utiliser votre main gauche durant votre enfance ?

- Oui ☐
- Non ☐

F12. Des membres de votre famille proche sont-ils gauchers?

	Oui	Non	Pas de réponse
Père	☐	☐	☐
Mère	☐	☐	☐
Frère	☐	☐	☐
Sœur	☐	☐	☐

	Oui	Non	Pas de réponse
Grands parents	☐	☐	☐
Frère ou sœur de votre père	☐	☐	☐
Frère ou sœur de votre mère	☐	☐	☐

F13. Quel est votre œil directeur (celui que vous utiliseriez pour regarder à travers un trou de serrure, par exemple) ?

Gauche ☐

Droit ☐

F14. Avez-vous un problème de vue concernant l'œil gauche ?

	Aucun problème	Léger	Moyen	Fort
Myopie	☐	☐	☐	☐
Astigmatie	☐	☐	☐	☐
Presbytie	☐	☐	☐	☐
Autre problème	☐	☐	☐	☐

F15. Avez-vous un problème de vue concernant l'œil droit?

	Aucun problème	Léger	Moyen	Fort
Myopie	☐	☐	☐	☐
Astigmatie	☐	☐	☐	☐
Presbytie	☐	☐	☐	☐
Autre	☐	☐	☐	☐

F16. Avez-vous un problème d'audition concernant l'oreille gauche?

Je n'ai pas de problème d'audition à gauche ☐

J'ai une déficience auditive légère (lorsque la perte moyenne est comprise entre 20 et 40 dB) ☐

J'ai une déficience auditive moyenne (lorsque la perte moyenne est comprise entre 40 et 70 dB) ☐

J'ai une déficience auditive sévère (lorsque la perte moyenne est comprise entre 70 et 90 dB) ☐

J'ai une déficience auditive profonde (lorsque la perte est supérieure à 90 dB) ☐

Autre ☐

F17. Avez-vous un problème d'audition concernant l'oreille droite ?

- [ ] Je n'ai pas de problème d'audition à droite
- [ ] J'ai une déficience auditive légère (lorsque la perte moyenne est comprise entre 20 et 40 dB)
- [ ] J'ai une déficience auditive moyenne (lorsque la perte moyenne est comprise entre 40 et 70 dB)
- [ ] J'ai une déficience auditive sévère (lorsque la perte moyenne est comprise entre 70 et 90 dB)
- [ ] J'ai une déficience auditive profonde (lorsque la perte est supérieure à 90 dB)
- [ ] Autre

F18. Avez-vous un problème de cervicales ?

- [ ] Je n'ai pas de problème de cervicales
- [ ] J'ai des douleurs/de la gêne dans le cou lorsque je tourne la tête vers la gauche
- [ ] J'ai des douleurs/de la gêne dans le cou lorsque je tourne la tête vers la droite
- [ ] J'ai des douleurs/de la gêne dans le cou lorsque je tourne la tête vers la gauche et vers la droite

F19. Avez-vous un problème de dos ?

- [ ] Je n'ai pas de problème de dos
- [ ] J'ai des douleurs/de la gêne lorsque je tourne le buste vers la gauche
- [ ] J'ai des douleurs/de la gêne lorsque je tourne le buste vers la droite
- [ ] J'ai des douleurs/de la gêne lorsque je tourne le buste vers la gauche et vers la droite
